# Targetable HER3 functions driving tumorigenic signaling in HER2-amplified cancers

**DOI:** 10.1016/j.celrep.2021.110291

**Published:** 2022-02-01

**Authors:** Marcia R. Campbell, Ana Ruiz-Saenz, Elliott Peterson, Christopher Agnew, Pelin Ayaz, Sam Garfinkle, Peter Littlefield, Veronica Steri, Julie Oeffinger, Maryjo Sampang, Yibing Shan, David E. Shaw, Natalia Jura, Mark M. Moasser

**Affiliations:** 1 Department of Medicine, University of California, San Francisco, San Francisco, CA 94143, USA; 2 Departments of Cell Biology & Medical Oncology, Erasmus Medical Center, Rotterdam, the Netherlands; 3 Cardiovascular Research Institute, University of California, San Francisco, San Francisco, CA 94143, USA; 4 D. E. Shaw Research, New York, NY 10036, USA; 5 Department of Biochemistry and Molecular Biophysics, Columbia University, New York, NY 10032, USA; 6 Department of Cellular and Molecular Pharmacology, University of California, San Francisco, San Francisco, CA 94143, USA; 7 Helen Diller Family Comprehensive Cancer Center, University of California, San Francisco, San Francisco, CA 94143, USA; 8 Lead contact

## Abstract

Effective inactivation of the HER2-HER3 tumor driver has remained elusive because of the challenging attributes of the pseudokinase HER3. We report a structure-function study of constitutive HER2-HER3 signaling to identify opportunities for targeting. The allosteric activation of the HER2 kinase domain (KD) by the HER3 KD is required for tumorigenic signaling and can potentially be targeted by allosteric inhibitors. ATP binding within the catalytically inactive HER3 KD provides structural rigidity that is important for signaling, but this is mimicked, not opposed, by small molecule ATP analogs, reported here in a bosutinib-bound crystal structure. Mutational disruption of ATP binding and molecular dynamics simulation of the apo KD of HER3 identify a conformational coupling of the ATP pocket with a hydrophobic AP-2 pocket, analogous to EGFR, that is critical for tumorigenic signaling and feasible for targeting. The value of these potential target sites is confirmed in tumor growth assays using gene replacement techniques.

## In brief

Campbell et al. undertake a structure-function analysis of the HER2-HER3 kinase domains when HER2 is overexpressed in cancers. The analysis confirms the critical role of this kinase dimer in this cancer type and identifies multiple new opportunities for the design of first-in-class allosteric inhibitors for this disease.

## Graphical Abstract

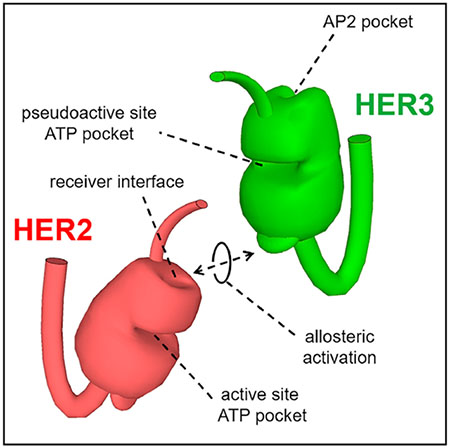

## INTRODUCTION

The treatment of cancers by targeting their driving kinase oncogenes is a highly rational and validated treatment approach, producing profound remissions in the majority of patients with targetable kinase oncogene-driven cancers, replacing cytotoxic chemotherapies (Chapman et al., 2011; Kantarjian et al., 2002; Maemondo et al., 2010; Shaw et al., 2013). However, the treatment of HER2-amplified breast cancers has not followed this paradigm, and HER2-targeting kinase inhibitors have minimal or modest activities by themselves (Blackwell et al., 2010; Burstein et al., 2008; Martin et al., 2013; Swaby et al., 2009). What sets HER2 apart is that it is activated by gene amplification and massive overexpression rather than mutation or fusion events. In addition, the critical role of its dimerization partner HER3 makes for a two-component tumorigenic driver, creating additional complexity in this target (Holbro et al., 2003; Lee-Hoeflich et al., 2008; Vaught et al., 2012). HER3 is not only essential for tumorigenesis but also functions in mediating drug resistance that becomes apparent when HER2 inhibitors are used to suppress HER2-HER3 signaling. This is because HER3 expression is highly dynamic and functionally linked with downstream negative feedback signaling, and the rapid compensatory upregulation of HER2-HER3 signaling following drug exposure allows for a 2-log increase in signaling output, presenting a stoichiometric barrier that has thus far remained outside the therapeutic index of all current pharmaceutical technologies, including best-in-class reversible and irreversible inhibitors acting at the active site of the HER2 kinase domain (KD) (Amin et al., 2010; Garrett et al., 2011; Sergina et al., 2007). There are HER2 isoforms, splice variants, and mutations in both human HER2-amplified cancers and in mouse Neu-driven tumors that show increased homodimerization and more aggressive biology (Hart et al., 2020; Siegel et al., 1994); however, HER3 remains an essential partner for tumorigenesis (Vaught et al., 2012). It has become clear that the highly effective treatment of HER2-amplified cancers requires combined inhibition of the functions of HER2 and HER3. But HER3 presents a challenging target for inhibition using current pharmaceutical strategies. Its function in HER2-amplified cancers is engaged in a ligand-independent manner, and antibodies targeting its extracellular domain (ECD) are unable to inhibit its signaling functions in these cancers (Blackburn et al., 2012; Garner et al., 2013; Kugel et al., 2014; McDonagh et al., 2012; Schoeberl et al., 2010; Campbell et al., 2022). Its KD is catalytically inactive and functions as an allosteric activator of the HER2 kinase (Guy et al., 1994; Jura et al., 2009b). This makes it much more challenging for functional targeting and is not suited for the standard pharmaceutical platforms for developing kinase inhibitors. Developing effective small molecule inhibitors of HER2-HER3 KD signaling requires deeper insights into the structural features of this complex that are engaged during tumorigenic signaling. We have performed a structure-function analysis of the HER2 and HER3 KDs to identify additional functional liabilities that can provide a mechanistic basis for next generation therapeutics.

## RESULTS

**Figure 1.**
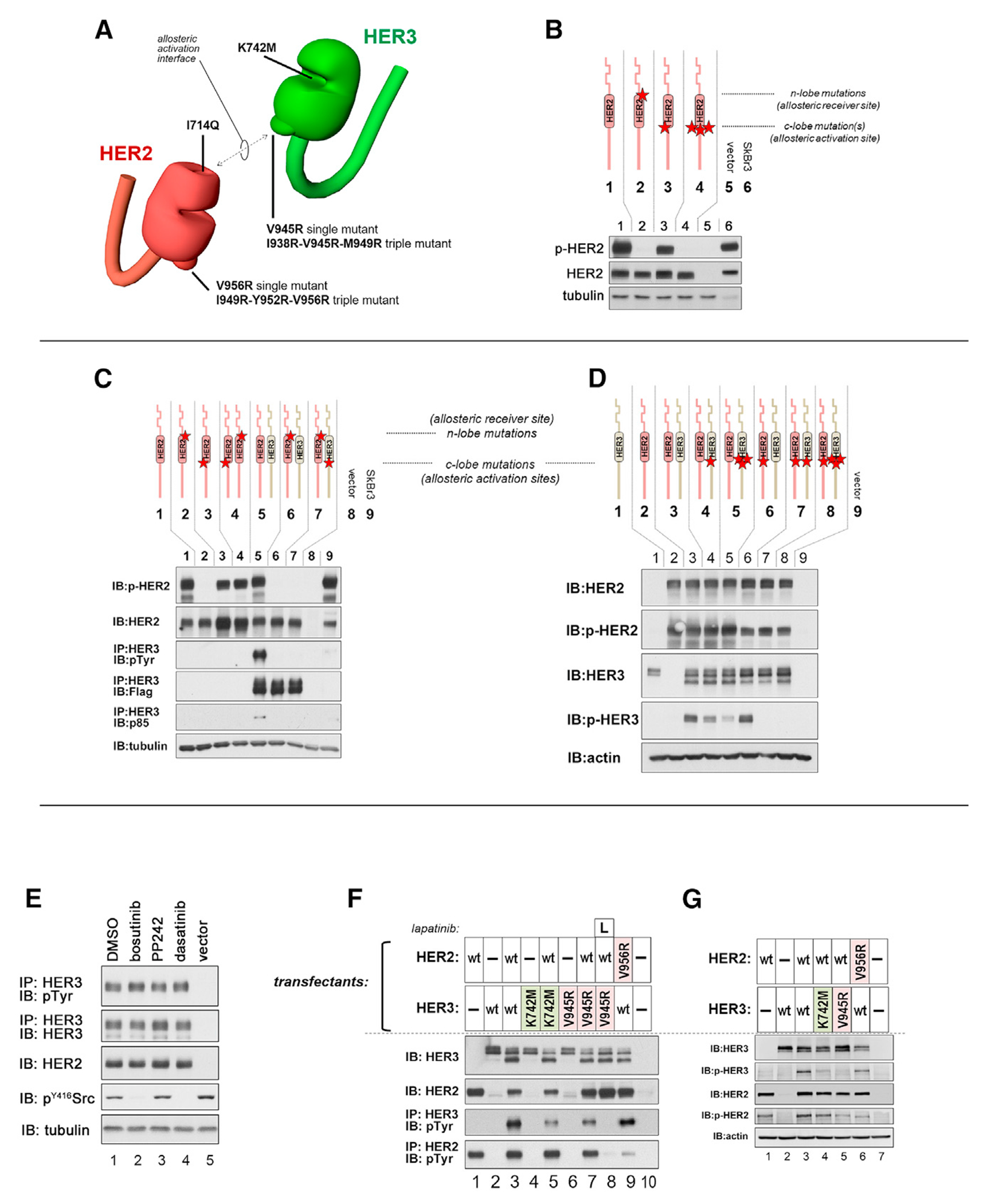
HER2 overexpression drives constitutive HER2-HER3 phosphorylation using the canonical allosteric activation mechanism and requires pseudoactive site occupancy in HER3 (A) Schematic depiction of the mutations drawn on cartoon representations of the kinase domains of HER2 and HER3. Amino acid (aa) numbering is based on the nascent reading frame. (B) CHO-K1 cells were cotransfected to overexpress HER2 and to express HER3, such as to generate constitutive HER2 autophosphorylation (lane 1) and HER3 transphosphorylation (lane 5). After 24 h, cell signaling was assayed as shown. Wild-type (WT) constructs were compared with mutants in the indicated combinations. The HER2 N-lobe mutant (I714Q) is a defective allosteric receiver, whereas the HER2 C-lobe mutant (V956R) is an impaired allosteric activator but competent receiver. HER3 is catalytically inactive and thus an obligate allosteric activator, and the HER3 C-lobe mutant (V945R) is an impaired allosteric activator. (C) CHO cells were transfected as in (B) using WT HER2 and HER3 in additional combinations. The HER2 C-lobe mutant (V956R) is an impaired allosteric activator. The HER3 single C-lobe mutant (V945R) is an impaired allosteric activator but retains some activation function. The HER3 triple C-lobe mutant (I938R-V945R-M949R) is an even more impaired allosteric activator. (D) CHO cells were transfected as in (B). The HER2 C-lobe activation function can similarly be further impaired by triple mutation. The double banding of HER3 observed in these CHO-K1 cell transfections is due to differential glycosylation (Figure S1B). (E) CHO cells were cotransfected to overexpress HER2 and express HER3 and were treated with DMSO or either of three small molecules that bind the HER3 KD with high affinity. These were administered at 1 μM for 1 h. The binding K_D_s for HER3 binding are as follows: bosutinib 0.8 nM, PP-242 120 nM, and dasatinib 18 nM (Davis et al., 2011). The affinities for HER2 KD are >1,400 nM for these drugs. The dephosphorylation of Src is shown as a positive control for bosutinib and dasatinib, which also inhibit Src. (F) CHO-K1 cells were transfected as in (A) using WT or mutant HER2 and HER3 constructs. The HER3 K742M mutant is defective at ATP binding. Lane 8 was treated with 1 μM lapatinib for 1 h. This image was cropped down from 13 lanes for clarity. (G) CHO-K1 cells were transfected as in (C) using WT or mutant HER2 and HER3 constructs.

We established an experimental system in CHO cells mimicking the expression levels and the ligand-independent constitutive phosphorylation of HER2 and HER3 that is observed in HER2-amplified cancer cells (Figure S1A). This was done because massive HER2 overexpression in cancer cells promotes ligand-independent modes of dimerization and signaling that is likely mechanistically distinct from canonical ligand-dependent modes of dimerization and physiologic signaling in cells with normal levels of HER2, and thus development of therapeutics based on the physiologic ligand-dependent modes often do not translate well to the pathologic ligand-independent signaling occurring in cancer cells. In the physiologic ligand-induced mode of signaling, the KDs of the HER family are activated through an asymmetric interaction wherein the C-lobe of an activator kinase interfaces with the N-lobe of a receiver kinase (Jura et al., 2009b; Kovacs et al., 2015; Qiu et al., 2008; Zhang et al., 2006). Constitutive HER2 autophosphorylation and HER3 transphosphorylation induced by the pathologic state of HER2 overexpression remain dependent on this allosteric mode of KD activation as seen by mutational disruption of this interface (Figures 1A–1D). The activating C-lobe interfaces are more difficult to disrupt by single mutation but are better disrupted by triple mutation (Figures 1B and 1D). All homotypic and heterotypic activation of the HER2 KD is channeled through its N-lobe receiver site such that mutation of this receiver interface results in total loss of HER2 and HER3 phosphorylation and signaling (Figures 1B and 1C). This N-lobe receiver site in the HER2 KD forms a hydrophobic groove potentially suitable for small molecule inhibitor binding (Figure S2).

The HER3 KD functions as an obligate allosteric activator and is itself catalytically inactive, despite high-affinity binding of ATP (Claus et al., 2018; Jura et al., 2009b; Shi et al., 2010). The role and relevance of ATP binding for the HER3 KD remains to be defined, but emerging evidence suggests a function in structural stabilization (Claus et al., 2018; Mendrola et al., 2013). We undertook an exploration of how occupation of the ATP pocket affects HER3 KD structure in this model of HER2-driven constitutive signaling. First, we determined whether displacement of ATP from HER3 KD by high-affinity-binding small molecule ATP analogs, all previously characterized as type I inhibitors, could have an effect. However, none of the small molecules suppressed constitutive HER3 signaling driven by HER2 overexpression (Figure 1E). Lack of the inhibitory effect of drug binding on HER3 signaling might be a consequence of the ability of these ATP-mimetic drugs to preserve the same conformation of the HER3 pseudokinase domain as ATP binding. This is supported by the effect of a K742M mutation in HER3, which disrupts ATP binding (Honegger et al., 1987; Shi et al., 2010), on HER3 phosphorylation (Figures 1F and 1G). In the presence of a K742M mutation, HER3 phosphorylation was significantly impaired under conditions of overexpression, to an extent comparable with the effect of V945R mutant that directly disrupts the allosteric function of the HER3 C-lobe. Hence the structural effect of ATP binding is critical for HER3 function even under conditions of HER2 overexpression. The function of HER3 impacts HER2 autophosphorylation, as well as HER3 transphosphorylation. HER2 autophosphorylation is higher if HER3 is present and competent to activate the HER2 KD compared with when HER2 autophosphorylation is driven entirely by homodimers (best seen in Figures 1C, 1F, and 1G), consistent with the notion that the HER3 KD is a better activator for the HER2 KD compared with the HER2 KD itself.

**Figure 2.**
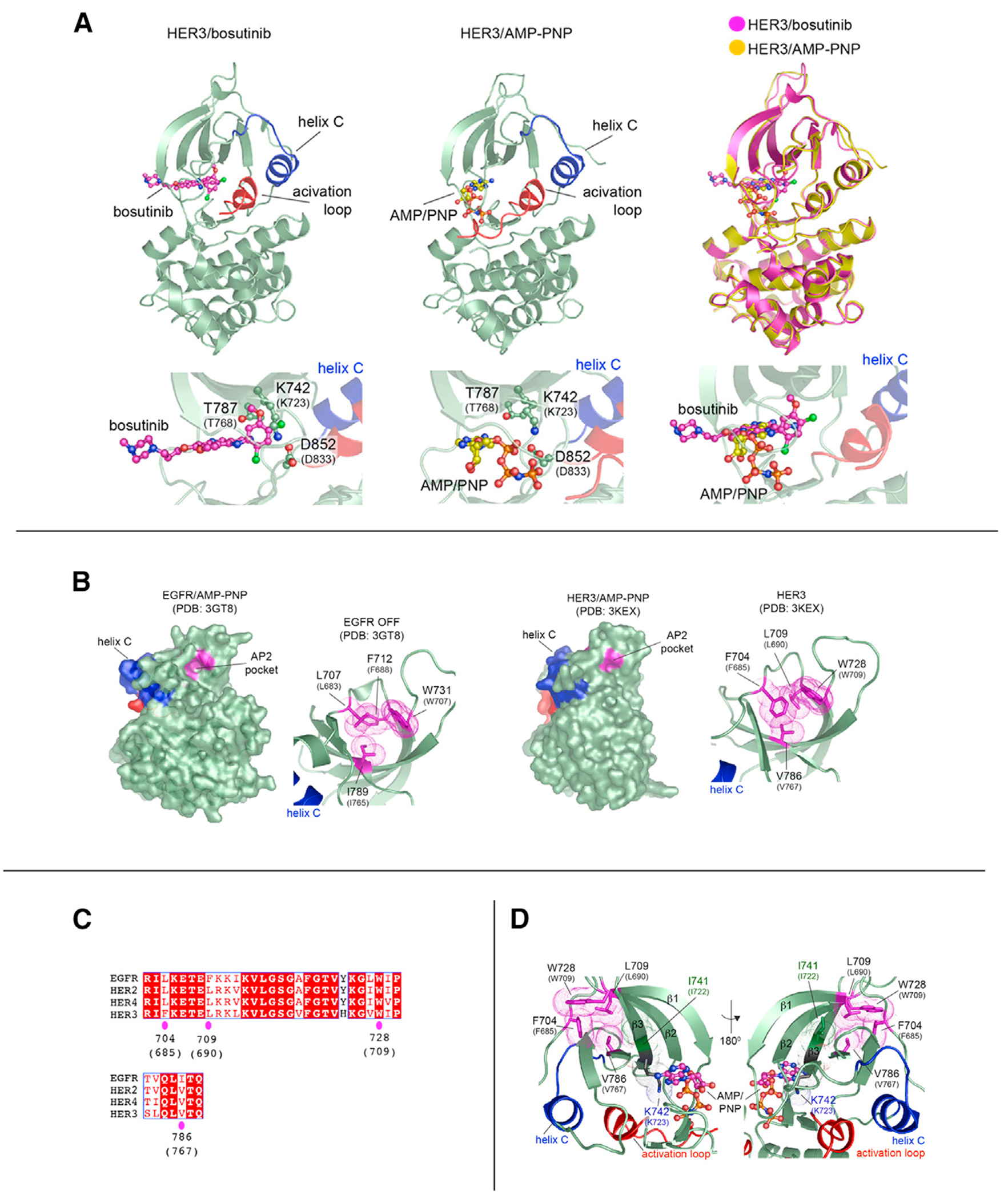
Bosutinib reinforces the Src/CDK-like conformation of the HER3 pseudokinase domain (A) Cartoon representation of crystal structures of HER3/bosutinib (PDB: 6OP9; left) and HER3/AMP-PNP (PDB: 3KEX; middle) and their overlay (right). Carbon atoms of bosutinib are shown in magenta; those of AMP-PNP are shown in yellow. The HER3/bosutinib complex adopts a Src/CDK-like inactive conformation in which helix C (blue) is rotated away from the active site, the activation loop (red) adopts a tethered conformation, and the DFG-aspartate (D852) adopts a DFG-in position. The methoxy substituent of bosutinib’s quinoline ring extends in the same direction as the ribose sugar of AMP-PNP, but the 2,4-dichloro-5-methoxyaniline fragment lies 4–8 Å away from the triphosphate linkage. Weaker electron density corresponding to the *N*-propoxy-*N*-methylpiperazine moiety indicated that this region of bosutinib does not interact strongly with HER3. T787 is a gatekeeper residue, and K742 is the “catalytic” lysine. Numbering corresponds to the nascent reading frame (top) or mature protein without the signal peptide (low, in parentheses). (B) The AP-2 pockets in the inactive EGFR kinase (PDB: 3GT8) and the HER3 kinase (PDB: 3KEX) are displayed in magenta and magnified in the panels to the right of full kinase domains shown in surface representation. Hydrophobic residues within the pockets are shown in a sticks and dot representation. Numbering corresponds to the nascent reading frame (top) or mature protein without the signal peptide (low, in parentheses). (C) Conservation of the AP-2 pocket residues in HER receptors. Magenta ovals show HER3 numbering. (D) The spatial relationship between the AP-2 pocket and the nucleotide-binding pocket in the HER3 kinase domain (PDB: 3KEX). K742 interacts with the V786 via hydrogen bond interactions within the β3/β4 strand. Side chains of I741 make direct hydrophobic interactions with V786 and L709.

To obtain deeper insights into the effects of drug binding to the HER3 KD, we determined the crystal structure of the HER3 KD/bosutinib complex to a resolution of 2.5 Å (Figures 2A and S3; Table S1). In this structure, HER3 KD adopts the so-called Src/CDK-like inactive conformation (Figure 2A), which is the same conformation that HER3 adopts when bound to the ATP analog AMP-PNP (root-mean-square deviation [RMSD] = 0.39 Å) (Jura et al., 2009b; Shi et al., 2010). The identical conformational states in the AMP-PNP and bosutinib-bound structures are achieved despite significantly different binding modes employed by bosutinib and the nucleotide (Figure 2A). This would support the notion that nucleotide pocket occupancy of HER3 plays an important structural role in HER3 signaling, and pocket vacancy is disruptive to the activating function of HER3.

Because there is no current pharmaceutical approach to induce a state of nucleotide pocket vacancy, we sought a deeper understanding of the structural effect that ATP binding might have on the conformation of the HER3 pseudokinase that may identify surrogate approaches more feasible for pharmaceutical targeting. The stabilizing effect of ATP is likely propagated through regions proximal to its binding site within the HER3 pseudokinase domain, and some of these are known to undergo conformational changes in related kinases. One such region located in the N-lobe of the KD is referred to as an AP-2 pocket in the epidermal growth factor receptor (EGFR) (Jura et al., 2009a). The AP-2 pocket adopts different conformations in the crystal structures of the inactive and active states of the EGFR KD. In the inactive conformation, the AP-2 pocket accommodates a portion of the EGFR C-terminal tail in a number of reported EGFR crystal structures (Figure S4A). In the structures of the active EGFR kinases, the C-terminal tail is displaced and the AP-2 pocket adopts a more closed conformation (Figures S4B and S4C). Similar conformational changes are observed in the Src tyrosine kinase in which the AP-2 pocket is engaged by the SH2-KD linker region in the inactive state and closes in the active state of the Src kinase (Breitenlechner et al., 2005; Xu et al., 1997). The physiological relevance of these interactions for kinase autoinhibition have been investigated for both EGFR (Jura et al., 2009a) and Src (Gonfloni et al., 1999).

**Figure 3.**
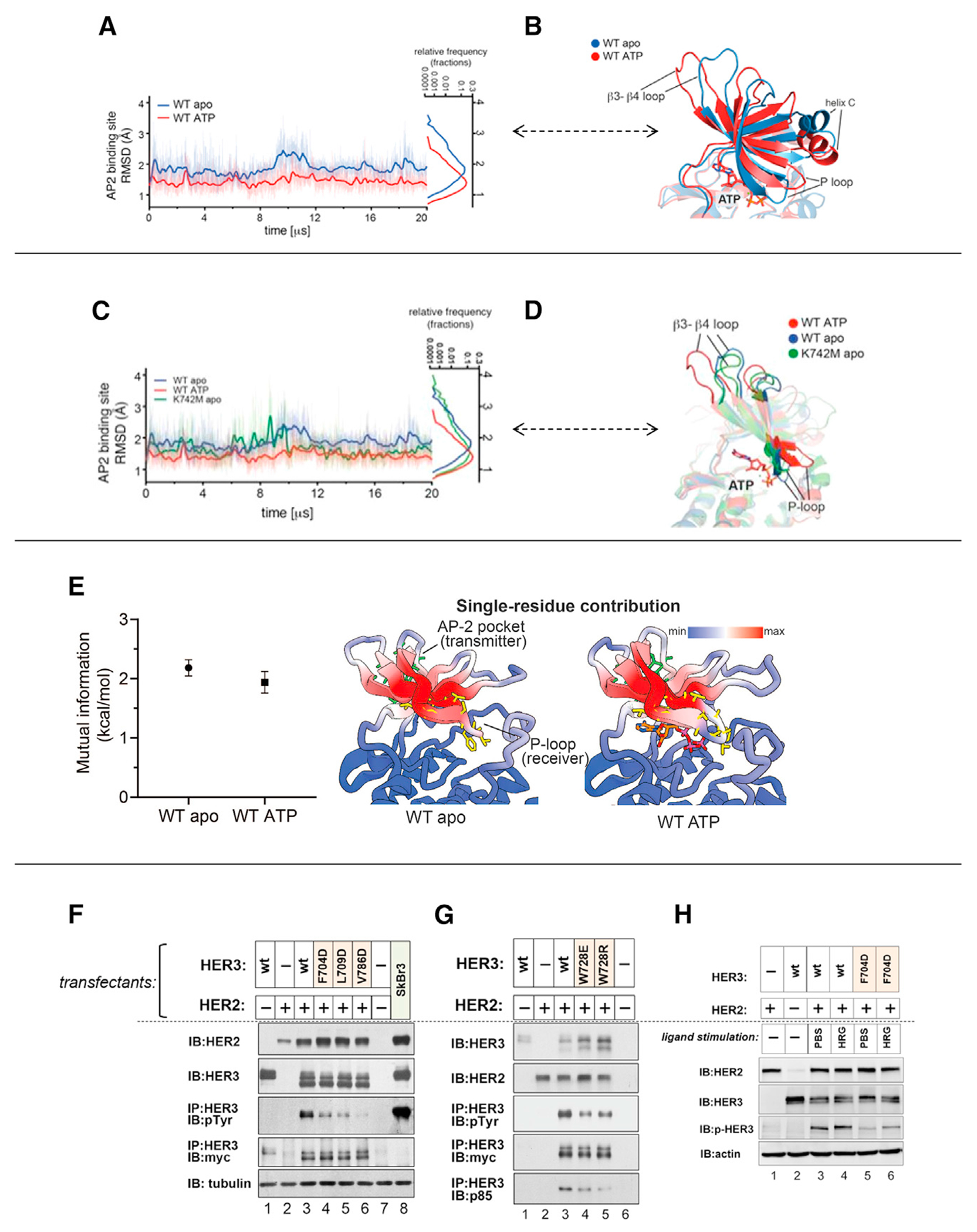
Conformation cross-talk between the surface AP-2 pocket of the HER3 KD and the pseudo-active site (A) RMSD plot from MD simulations of the ATP-bound and apo HER3 pseudokinase states. (B) The superimposed cartoon images of the ATP-bound and apo HER3 final pseudokinase states showcase inward movement of the β3-β4 loop and the collapse of the P loop in the apo state. (C) RMSD plot from MD simulations of the ATP-bound WT, apo WT, and apo K742M HER3 pseudokinase states. (D) The superimposed cartoon images of the ATP-bound WT, apo WT, and apo K742M HER3 final pseudokinase states showcase inward movement of the β3-β4 loop and the collapse of the P loop in the apo state. (E) Left: plot of mutual information in kcal/mol between the transmitter (AP-2 pocket: residues 683, 685, 689, 690, 709, 718, 720, 756, and 767; side chains shown in green in right panel) and receiver (P loop: residues 696–704; side chains shown in yellow in right panel) sites, showing ~2 kcal/mol mutual information in both the absence (WT apo) and presence (WT ATP) of ATP. Error bars reflect standard deviation. Right: illustration of the normalized per-residue contribution to co-information in apo and ATP-bound simulations of HER3; “min” and “max” refer to the minimum and maximum values among all possible residues across all simulations. Residues colored dark red contribute most to communication between the AP-2 pocket and the P loop. (F) CHO-K1 cells were transfected to overexpress HER2 and to express the indicated WT or mutant HER3 constructs. The surface residues F704, L709, and V786 were mutated to impair the binding affinity of the AP-2 pocket. (G) W728 was mutated to collapse the AP-2 pocket. (H) The effect of ligand stimulation was studied on the F704 AP-2 pocket mutant.

The AP-2 pocket is largely conserved between EGFR and HER3, and it encompasses four hydrophobic residues in HER3: F704, L709, W728, and V786. The pocket adopts a partially open conformation in both the ATP- and bosutinib-bound HER3 structures, stabilized by an edge-to-face π-stacking interaction between F704 and W728 (Figures 2B, 2C, and S5). Most importantly, it is connected to K742 in the nucleotide-binding pocket via a network of hydrogen bonds within the β3/β4 strand that extends to V786 in the AP-2 pocket. The side chain of the preceding residue, I741, makes direct hydrophobic interactions with the side chains of L709 and V786 (Figure 2D). We used molecular dynamics (MD) simulations to investigate the dynamics of the AP-2 pocket as a function of the nucleotide occupancy in the ATP binding site. The AP-2 residues in the ATP-bound HER3 pseudokinase domain remained largely stable throughout the length of the simulation with an average RMSD of 0.6 Å (Figures 3A and 3B). In contrast, apo HER3 and K742M HER3 mutant underwent larger fluctuations within the N lobe and in the AP-2 pocket in particular (Figures 3A–3D). These fluctuations were accompanied by a significant downward shift of the β3-β4 loop in the AP-2 pocket and the collapsed conformation of the P loop in the pseudo-active site (Figures 3B and 3D). The collapsed P loop state likely reflects loss of stabilizing interactions provided by the phosphate groups of the bound nucleotide. With the exception of the conformational changes centered on the nucleotide-binding pocket, there were no other structural rearrangements that were significantly different between the ATP-bound and apo HER3 or HER3 K742M mutant. Hence what we observe is a specific “seesaw” motion of the structural elements spanning from the pseudoactive site to the AP-2 pocket, pointing to an allosteric connection between these two sites in the HER3 pseudokinase.

To investigate whether there is allosteric coupling between the AP-2 pocket and the ATP binding site, we performed an N-body Information Theory (NbIT) (LeVine and Weinstein, 2014) analysis on MD simulations of ATP-bound and apo HER3 (Figure 3E). In this analysis, we treated as the receiver site the residues of the P loop, the region of the ATP binding site farthest from the AP-2 pocket, which is an important component of the ATP binding site due to its involvement in mediating binding and transferring the terminal phosphate group of ATP. The NbIT analysis demonstrated allosteric communication in our simulations between the AP-2 pocket and the P loop (~2 kcal/mol), providing further support for the notion that the AP-2 pocket should be considered as a target site for the design of drugs that could affect the binding of molecules within the ATP binding site.

The function-disabling effect of ATP loss on HER3 induced by the K742M mutation (Figures 1F and 1G) can be mimicked by direct mutation of the AP-2 pocket, either through mutation of W728, which would collapse the AP-2 pocket, or any of three surface residues (F704, L709, V816) that could interfere with the potential binding activities of the pocket (Figures 3F and 3G). All of these mutations compromise HER3 signaling to an extent observed for the K742M and V945R mutations. In the face of HER2 overexpression simulated in these experiments, constitutive ligand-independent signaling is quite high, and there is only an incremental increase in phosphorylation inducible by ligand. Both the constitutive and ligand-induced levels of signaling are considerably decreased by an AP-2 pocket mutation (Figure 3H). These data further support the notion that the ATP binding pocket and the AP-2 pocket are in the same allosteric path, the integrity of which is essential for HER3 function.

In contrast to active kinases, pseudokinases typically do not sample many conformational states that could be preferentially stabilized by diverse classes of molecules occupying the ATP pocket (Murphy et al., 2017). But the structural rigidity offered by the occupants of this pocket remains paramount. Exploring this requirement through these experimental *in vitro* and *in silico* ATP eviction studies identifies the surface AP-2 pocket that is in conformational cross-talk with the ATP pocket. Similar to disruption of ATP binding in the pseudoactive site of HER3, mutation of the AP-2 pocket has an inhibitory effect on HER3 signaling, providing an alternative binding site for targeting the signaling functions of HER3. To evaluate the potential of the AP-2 pocket to engage small molecules (Shan et al., 2011), we conducted unbiased fragment binding MD simulations with randomly selected fragments containing aromatic rings and observed multiple transient binding events in the AP-2 pocket (Figure S6). Although more systematic MD studies are required to identify “hit” binders of the pocket, the simulations in addition to the structural studies on EGFR and Src kinases that demonstrate the capacity of this pocket to engage in binding of short polypeptide motifs (Gonfloni et al., 1999; Jura et al., 2009a) (Figure S6) underscore the potential for binding small molecules by the AP-2 pocket.

**Figure 4.**
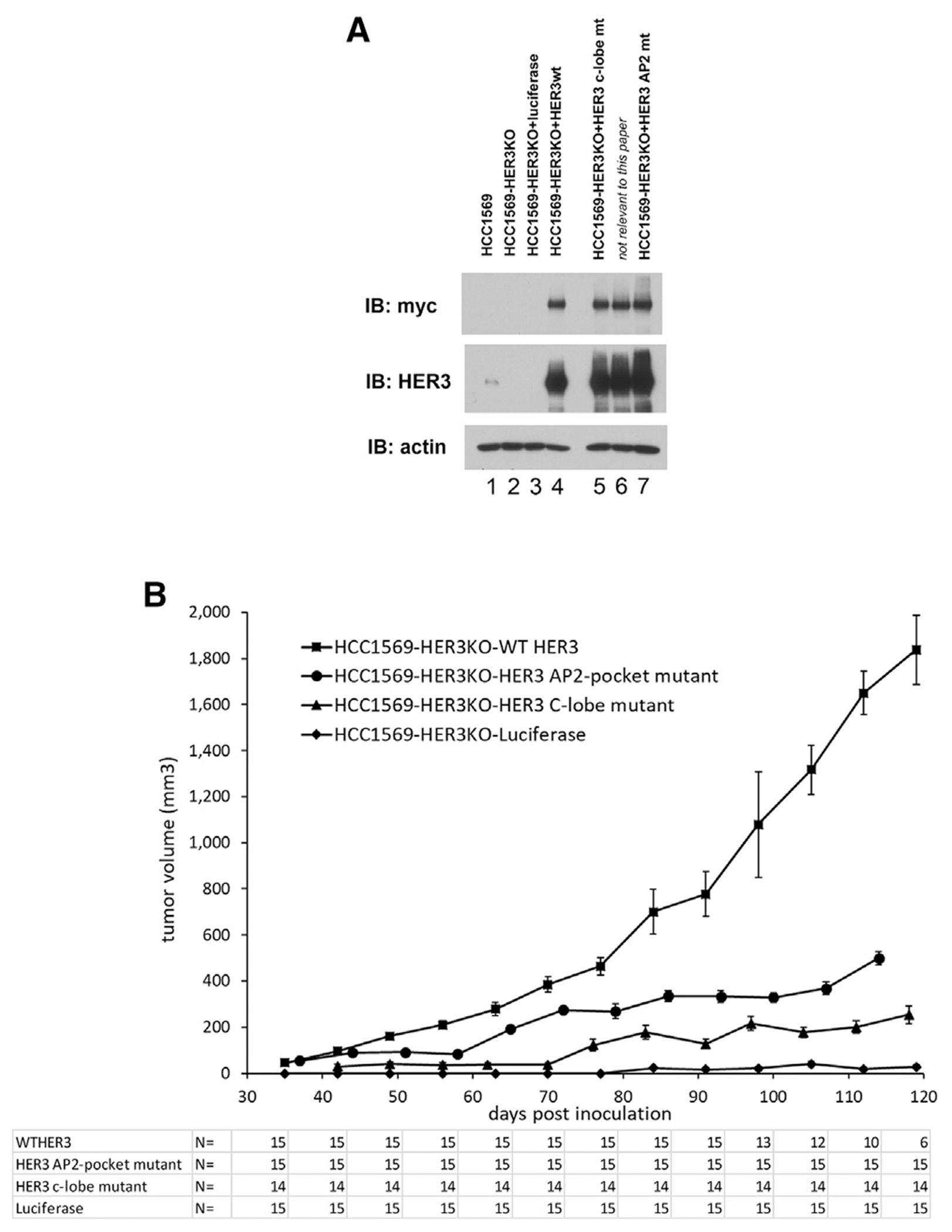
HER2-HER3 allosteric transactivation and HER3 AP-2 pocket functions are required for the growth of HER2-amplified tumors *in vivo* (A) HCC1569 human HER2-amplified breast cancer cells were engineered to eliminate HER3 expression by CRISPR-Cas targeting (HCC1569-HER3KO). To eliminate the role of clonal growth characteristics in the replacement experiments, we mixed together three separate clones of HCC1569-HER3KO cells to generate a polyclonal HCC1569-HER3KO cell line (lane 2), and this cell line was used as the parental cell line for the various add-back experiments. These were then transduced to re-express WT HER3 (lane 4) or experimental mutant HER3 constructs. Experimental add-backs included a HER3 C-lobe mutant defective at allosteric activation (HER3 I938R/V945R/M949R) and a HER3 with a mutated AP-2 pocket (F704D). The expression of firefly luciferase (lane 3) constitutes a negative control cell type. The add-back HER3 constructs contain C-terminal myc tags. (B) The indicated engineered versions of HER2-amplified HCC1569 tumor cells were inoculated subcutaneously in NSG mice, and tumor volumes were measured over time. The number of surviving mice along the time course of the animal studies is shown for each arm underneath, and the sample size reduction over time in some arms reflects the removal of mice for euthanasia because of large tumor sizes as mandated by guidelines. The error bars reflect SEM.

The importance of these KD functions within the HER3 KD on tumorigenic growth was confirmed in HCC1569 HER2-amplified cancer cells. Although the CHO cell studies described above allow highly controlled structure-function studies to be performed in isolation from confounding variables, the HCC1569 *in vivo* studies allow the testing of these conclusions in an actual HER2-amplified tumor, accounting for all the complexities of cancer cell signaling, as well as any contributions from the *in vivo* microenvironment, including the potential role of *in vivo* ligands. To generate this model, we eliminated the endogenous expression of HER3 in these cells by CRISPR-Cas9 targeting and replaced it with the expression of experimentally controlled HER3 constructs, including wild-type HER3 or versions carrying mutations at the c-lobe interface or at the AP-2 pocket (Figure 4A). Disruption of the allosteric activating function within the HER3 KD C-lobe or disrupting the AP-2 helix binding activity of the HER3 KD N-lobe substantially impairs tumor growth (Figure 4B), confirming the essential role of these functions in tumorigenic signaling and the potential of these sites as targets for novel classes of pharmaceutical agents. It is unlikely that the engineered mutations in this study, including the HER3 C-lobe activating interface mutations and the HER3 AP-2 pocket mutations, are globally disrupting the entire structural integrity of the HER3 KD and producing an entirely nonfunctional or unfolded protein. These mutations are at surface residues, mutations at different residues produce similar results, the mutant versions of HER3 are stable proteins, they express as well as the wild-type HER3 (Figures 1B–1G, 3G, 3H, and 4A), and they localize properly within cells (Figure S7). The tumor-suppressive effects of these mutations are consistent with the structural and biochemical evidence presented, supporting a specific loss of function inflicted by these targeted mutations.

## DISCUSSION

The treatment of oncogene-driven cancers through the inhibition of their driving kinase oncogenes has revolutionized the field of oncology, and this treatment paradigm has now replaced cytotoxic chemotherapeutics in the clinical management of many oncogene-driven cancers (Chapman et al., 2011; Kantarjian et al., 2002; Maemondo et al., 2010; Shaw et al., 2013; Verweij et al., 2003). This success to date has been largely restricted to kinases activated through mutational or fusion events. These same drugs that are effective against mutation- or fusion-activated kinases are far less effective when the same kinase target is activated through gene amplification and overexpression (Barnes et al., 2005; Campbell et al., 2002; Cohen et al., 2005; Corcoran et al., 2010; Hodi et al., 2013; Landi et al., 2019; Nukaga et al., 2017; Soulieres et al., 2004). This includes inhibitors of HER2, which are far more effective in treating cancers driven by mutational activation of HER2 (Hyman et al., 2018) than cancers driven by amplification and overexpression of HER2 (Burstein et al., 2010; Martin et al., 2013). It is evident that massively overexpressed kinase oncogenes present a barrier that has yet to be overcome. That is not altogether surprising because the stoichiometry of kinase inhibition is altered by sheer abundance. Theoretical approaches to overcome this include a similar massive increase in drug dosage and exposure, and we attempted such an approach (Chien et al., 2011), but this is limited by the therapeutic index and bioavailability of the agents. In another approach, irreversible kinase inhibitors can provide substantially increased molar potency afforded by covalent binding to the KD, and this superiority is readily evident *in vitro*. But such chemical reactivity comes at a significant cost in expansion of off-targets, including targets within (Davis et al., 2011; Karaman et al., 2008) and outside of the kinome family (Dittus et al., 2017; Lanning et al., 2014; Niessen et al., 2017), and thus the increased biochemical potency of these agents is offset by their limited therapeutic index. Clearly, additional approaches are needed to effectively inhibit massively overexpressed driver kinases.

Of the overexpressed kinase oncogenes, HER2 amplification and overexpression account for the largest subset of cancers. The HER2 target brings with it additional complexity in the form of its requisite dimerization partner HER3, which is reviewed in the Introduction. Although the expression of HER3 is far less than HER2 in these tumors, the expression of HER3 is dynamic, and HER2-HER3 signaling output is increased nearly 100-fold through the compensatory upregulation of HER3, unleashing a great reserve capacity inherent in the massively abundant HER2 (Amin et al., 2010; Sergina et al., 2007; Garrett et al., 2011). In this structure-function study, we looked for alternative mechanistic approaches to interfere with HER2-HER3 transactivation that can complement current ATP analog HER2 kinase inhibitors, increasing the potency of target inhibition. We specifically modeled the state of massive overexpression in these experimental studies because overexpression may involve mechanisms not engaged during physiologic ligand-driven receptor dimerization and activation, which occur at normal levels of expression.

Although high-resolution structures of the HER2 KD homodimer or a HER2-HER3 KD heterodimer have not yet been resolved, the activating interaction between the HER2 N-lobe receiver site and the HER3 C-lobe activator site can be modeled based on the crystal structures of the active EGFR and HER4 kinase homodimers or HER3 kinase/EGFR kinase heterodimers (Littlefield et al., 2014; Qiu et al., 2008; Zhang et al., 2006). Our data show that mutations at the dimer interface that target either the receiver interface on HER2 or the activator interface on HER3 efficiently disrupt HER2/HER3 signaling under conditions of HER2 overexpression. Hence our findings suggest a potential in allosteric inhibitors acting at the active dimer interface as an efficient strategy in tumors driven by HER2 overexpression. Design of such molecules is theoretically possible on the HER2 side. The HER2 interface at the HER2/HER3 asymmetric kinase dimer centers on a so-called helix αC patch, which is a hydrophobic pocket formed by the αB and αC helices and the β4/β5 strands in the N-terminal lobe of the KD (Figure S2). The helix αC patch serves as a regulatory allosteric site in a number of protein kinases (Jura et al., 2011). In the PDK1 kinase, the helix αC patch has been successfully targeted by small molecule inhibitors (Engel et al., 2006; Rettenmaier et al., 2014), setting a precedence for this strategy working in other kinases. In contrast, finding molecules that target the asymmetric kinase dimerization interface on the HER3 side will not be trivial because this side of the active dimer interface lacks suitable pockets that could be explored for targeting. Our data suggest two alternative sites on the HER3 KD that, although not directly at the dimer interface, form well-defined pockets and whose structural integrity is essential for HER2/HER3 signaling. First is the canonical nucleotide-binding pocket that is preserved in HER3 despite the fact that HER3 is a pseudokinase receptor and does not have the ability to hydrolyze ATP (Jura et al., 2009b). Disruption of ATP binding in HER3 by mutation has previously been shown to inhibit its signaling (Claus et al., 2018), and we further demonstrate that this requirement cannot be overcome by massive HER2 overexpression as seen in HER2-amplified cancers. These data point to an essential structural role of nucleotide coordination for signaling by the HER3 pseudokinase, a phenomenon noted in other pseudokinases, such as STRADalpha, KASR, and JAK2 (Hammaren et al., 2015; Hu et al., 2011; Zeqiraj et al., 2009). These observations also suggest that there may be a potential in very specifically designed molecules binding within the ATP pocket of the HER3 KD to serve as its inhibitors, if these molecules can displace ATP without satisfying structural interactions made by the ATP.

Alternative approaches to destabilize the ATP pocket of the HER3 KD include targeting sites involved in structural cross-talk with this pocket. In this effort we identify one such site, the AP-2 pocket on the N-lobe of HER3 KD, with mutational studies that support its relevance even in the face of massive HER2 expression. We originally identified the AP-2 pocket in the EGFR KD, although its exact functions remain to be well defined. This pocket engages in protein-protein interactions that have been previously identified to occur inter-molecularly in EGFR. Specifically, we had shown that the AP-2 pocket of one EGFR monomer engages the C-terminal tail fragment of another EGFR receptor resulting in formation of an autoinhibited complex (Jura et al., 2009a). These observations had revealed the potential of the AP-2 pocket to engage in binding events that hypothetically could be explored for drug targeting. Although at present, we do not know if the AP-2 pocket in HER3 can also engage an AP-2 helix region, the conservation of the pocket among all HER receptors supports this possibility. If it does, the AP-2 helix region must come from another HER receptor (e.g., HER2) because the AP-2 helix sequence is not conserved in HER3 (Jura et al., 2009a). It is also possible that other motifs engage with the AP-2 pocket of HER3 because in the Src kinase, the AP-2 pocket engages a structurally different SH2 domain-KD linker region (Gonfloni et al., 1999). Although the functionally important role of the HER3 AP-2 pocket for HER3 and its dimers with HER2 is unclear, the disruptive effect of AP-2 pocket mutations on HER2/HER3 signaling suggests the importance of the AP-2 pocket structural integrity for HER3 signaling. The mechanisms by which the putative small molecule binders of the AP-2 pocket might disrupt HER3 signaling remain speculative. Our data suggest that these compounds could signal to the ATP site and induce conformational changes resulting in the dissociation of the nucleotide from the active site.

Agents targeting the receiver interface of HER2 or the AP-2 pocket of HER3 may not have the level of potency that can phenocopy the effects seen with engineered mutations at these sites. But the activities of such agents would be expected to combine favorably with current HER2 kinase inhibitors, and the combination may provide the requisite potency to fully and durably inhibit HER2-HER3 signaling in HER2-amplified cancers, a task that is beyond the therapeutic index of current modalities. Indeed, combinations strategies are most likely needed to effectively inactivate massively overexpressed kinases, such as HER2. The highly suppressed nature of the engineered HER3-mutant tumors precludes the conduct of informative lapatinib combination studies, and predicting the efficacy of such combinations must await the development of prototype compounds, which can be titrated in combination studies. Combinations with extracellular domain (ECD) targeting biotherapeutics have and will continue to be explored, although that effort crosses into mechanisms beyond inhibition of oncogenic signaling. Much of the clinical progress to date in the treatment of HER2-amplified cancers has been on the shoulders of these classes of agents, including HER2-targeting antibodies and HER2-targeting antibody-drug conjugates. The clinical activity of these agents is mediated through immunologic mechanisms or through the targeted delivery of cytotoxic agents, and continued improvements in these mechanistic approaches are underway. However, there is little disruption of HER2-HER3 signaling afforded by targeting the ECDs in HER2-overexpressing tumor cells (Campbell et al., 2022), and these approaches pursue a different mechanistic path.

### Limitations of the study

This study identifies potential allosteric sites for the design of small molecules that could disrupt HER2-HER3 signaling, and the importance of these sites and their functional relevance is confirmed by mutational studies. However, it remains unknown whether small molecules that can bind these sites with sufficient affinity and selectivity can be identified. The simulations of fragment engagement with the HER3 AP-2 pocket shown in this study are admittedly speculative. Only a concerted drug discovery effort can establish the druggability of these sites and their translational potential. In this study, we have interrogated the functions of allosteric sites through mutational studies and have shown that these engineered mutations are not destabilizing the receptors as best as can be determined through expression levels and localization properties. However, it remains possible that engineered mutations can induce unexpected or undesired conformational changes, and the functions of the receptors could be altered in a fashion broader than the intended narrow effects.

## STAR★METHODS

### RESOURCE AVAILABILITY

#### Lead contact

Further information and requests for reagents should be directed to and will be fulfilled by the lead contact, Mark Moasser (mark.moasser@ucsf.edu).

#### Materials availability

Plasmids generated in this study are available from the lead contact upon request. The modified cell lines are available from the lead contact upon request under a material transfer agreement. The molecular dynamics trajectories described in this work are available for non-commercial use through contacting trajectories@deshawresearch.com.

#### Data and code availability

- Data reported in this paper will be shared by the lead contact upon request. The Protein Data Bank (PDB) accession code for the crystallographic structure reported in this paper is 6OP9 and the data is available at https://www.rcsb.org/structure/6OP9.
- The molecular dynamics (MD) simulations were performed using the Anton 2 supercomputer. The simulation code we used is specialized to Anton 2, but codes for performing MD simulation are widely available. The molecular dynamics trajectories described in this work are available for non-commercial use through contacting trajectories@deshawresearch.com.
- Any additional information required to reanalyze the data reported in this paper is available from the lead contact upon request.

### EXPERIMENTAL MODEL AND SUBJECT DETAILS

#### Animal studies

All animal experiments were approved by the Institutional Animal Care and Use Committee at UCSF (IACUC).

### METHOD DETAILS

#### Cell culture

All cells (HCC1569, SkBr3, and CHO-K1) were maintained at 37°C and 5% CO2. HCC1569 cells were grown in RPMI1640 media, SkBr3 in DMEM Hams:F12 media, and CHO-K1 cells in F12K media. All media was supplemented with 10% fetal bovine serum, penicillin, streptomycin, and L-glutamine. HCC1569 M1 cells are a subclone of HCC1569 that were obtained after passage of HCC1569 cells in the mammary fat pad of nude mice and reestablishment in monolayer culture. For use in *in vitro* experiments lapatinib was purified from tablets (Glaxosmithkline) as previously described (Amin et al., 2010).

#### Protein lysate preparation and immunoblotting

Cells were lysed in modified RIPA (mRIPA) buffer (1% Na Deoxycholate, 0.1% SDS, 1% NP-40 detergent, 150mM NaCl, 10mM Na phosphate buffer) supplemented with leupeptin, aprotinin, phenylmethylsulfonyl fluoride, sodium vanadate and a phosphatase inhibitor cocktail, incubated on ice for 30 min and precleared at 14,000 rpm for 10 min at 4°C. Lysates were quantified using the Pierce BCA assay. 30-50ug of protein lysate was denatured by boiling for 8 min with laemmli sample buffer and separated on 7-10% polyacrylamide gels and transferred to PVDF membrane at 100V for 1.5 h in the cold room. Membranes were blocked in 3% bovine serum albumin (BSA) for 45 min and immunoblotted overnight at 4°C with the relevant primary antibodies and visualized using horseradish peroxidase conjugated secondary antibodies. These transfection assays and western blots have been performed multiple times and results found to be reproducible.

#### Transfection of CHO-K1 cells

To recapitulate the disease state of HER2 overexpression we used a CHO cell transient transfection model system. 10cm petri dishes were seeded with 3.5 X106 cells and transfected 24 h later. A total of 7ug of DNA (4.5ug of HER2 and 2.5ug of HER3) and 21ul of Lipofectamine 2000 were combined according to manufacturer’s directions and added to cells that had been washed with PBS and primed with 3.5mL optimem serum free media. After a 4 h incubation at 37°C the transfection complexes were replaced with fresh F12K media (with/without 10% FBS depending on the experiment). The next day cells were either treated with drug and harvested or immediately lysed into 375-425ul of cold modified RIPA buffer.

#### Immunoprecipitation

For immunoprecipitation of exogenously expressed proteins from CHO-K1 cells, cells were washed 1 X with PBS, lysed in mRIPA lysis buffer (400ul/10cm plate) so that the final concentration of proteins was between 2-5ug/ul. 150-600ug of protein lysate from each sample was brought up to a common volume, incubated with the targeting antibody. The mix was rotated overnight at 4°C and the following day immunoprecipitated using either Protein G Sepharose 4 fast flow beads or Streptavidin conjugated agarose beads at 4°C for 1-2 h. The protein-antibody-bead complexes were pelleted at 8000rpm for 2 min and washed with cold modified RIPA buffer three times. The precipitated complexes were resuspended in 35ul of 2 X laemmli buffer, boiled for 8-10 min and separated on polyacrylamide gels. Immunoblotting was done using previously described antibodies.

#### Immunofluorescence assays

CHO-K1 or HCC1569 cells were seeded onto glass cover slips in 12 well plates that had been coated with 1ug/ml of fibronectin and dried. The next day cells were transfected with the relevant HER3 constructs tagged with C-terminal SNAP tags and allowed to recover overnight. 24 h following transfection cells were washed in PBS and stained with SNAP-Cell 647-SiR. Following the SNAP-labeling substrate incubation, the cells were fixed for 10 min with 4.0% paraformaldehyde at room temperature. The cells were washed with PBS, incubated in 5.0 μM Hoechst 33342 (as a nuclear counterstain) for 2 min, and washed again in PBS. The coverslips were gently dried and mounted onto glass microscope slides using ProLong Gold antifade reagent (without DAPI). The slides were protected from light and allowed to cure for 24 h prior to imaging. Slides were prepared for long-term storage by sealing the coverslips with clear nail polish and storing at −20°C.

#### DNA plasmid construction

Vector cloning was done using Gateway Cloning technology. HER2 and HER3 ORFs were cloned into entry vectors from Thermo-FisherScientific (pDONR221 or pENTR4). Additionally we used pDONR223-HER3 (ERBB3) which was a gift from William Hahn & David Root (Addgene plasmid # 23874; http://n2t.net/addgene:23874; RRID:Addgene_23874). Some mutant constructs were generated using mismatched primers and mutation-specific PCR conditions. Other constructs were generated using gene synthesis (Genewiz). Destination vectors used were pcDNA-DEST40 (contains c-terminal V5-His tags), or modified versions of this vector to express c-terminal 2XFlag tags (pcDNA-DEST40-2XFlag) or 2XHA (pDNA-DEST40-2XHA) tags. Lentiviral infections were done using pLEX-ires-GFP, modified from pLEX_307 destination vector (gift from David Root; Addgene plasmid # 41392 ; http://n2t.net/addgene:41392 ; RRID:Addgene_41392). The HER3-ECDΔgLuc construct consists of the entire Gaussia Luiferase ORF fused to a c-terminal 10AA GGS linker and fused to a truncated HER3 consisting of AAs 642-1342 encompassing the HER3 transmembrane and entire intracellular domains.

#### HCC1569 HER3 gene switch cell line generation

The deletion of HER3 in HCC1569 breast cancer cells was previously described (Ruiz-Saenz et al., 2018). Briefly, using CRISPR-Cas9 technology, we engineered the elimination of HER3 expression in HER2-amplified HCC1569 breast cancer cells. Three independent HER3 knockout (HCC1569-HER3KO) clones were confirmed to lack HER3 protein expression and were selected for further analysis. These HCC1569KO cells maintain proliferative growth in monolayer cell culture, but are substantially deficient in tumorigenesis in mouse xenograft hosts. To eliminate the role of clonal growth characteristics in the replacement experiments, the three separate clones of HCC1569-HER3KO cells were mixed together to generate a polyclonal HCC1569-HER3KO cell line and this cell line was used as the parental cell line for the various add-back studies described in this paper.

To generate the various HER3 add-back cell lines, HCC1569-HER3KO cells were transduced with wild-type or mutant versions of HER3. Mutant versions of HER3 were cloned into Gateway entry vectors and shuttled into the pLEX-ires-eGFP destination vector using lentivirus particles produced, concentrated and titered at the UCSF lentiviral core (https://viracore.ucsf.edu/). Briefly, viruses were produced in 10cm petri dishes using jetPRIME transfection reagent and 3rd generation packaging plasmids. 72hrs post transfection, viral supernatants were collected, filtered, concentrated, titered and frozen immediately at −80°C. Due to the very large size of the HER3 pLEX lentiviral plasmids (13.9 kB), care was taken when handling viral supernatants. For viral transduction, HCC1569HER3KO cells were seeded into one well of a 24-well plate. The next day cells were refreshed with fresh media for 6 h before transduction. Virus was thawed at room temperature and brought up to 700ul with RPMI media. Virus with 1 X Transdux reagent was added to cells and incubated overnight at 32°C. The following day 300ul of fresh media was added and cells were transferred to 37°C for 6 h. At the end of the day the virus containing mix was replaced with fresh RPMI media. 96-120hrs post transduction, cells were selected with 1ug/ml of puromycin for at least 2 weeks. Our pLEX lentiviral construct contains the puromycin resistance gene as well as an IRES eGFP viral backbone. Fluorescence activated cell sorting was used to isolate and pool eGFP positive cells and exogenous HER3 expression was confirmed by western blotting of the Myc-His tag. Pooled cells were grown in 0.25ug/ml puromycin to maintain exogenous HER3 expression. The re-expression of HER3 in HCC1569HER3KO cells restores tumorigenic growth, although with a slower growth rate than the parental HCC1569 cells.

#### Mouse tumor growth assays

All animal experiments were approved by the Institutional Animal Care and Use Committee at UCSF (IACUC). A total of 5X106 HCC1569 M1 cells in 100ul (50% matrigel:50% serum free media) were implanted subcutaneously or into the mammary fat pad (where indicated) into NSG mice. Tumor growth was measured weekly starting about 4 weeks post cell implantation or when tumors became large enough to measure. When tumors reached the maximum size allowed under our IACUC guidelines, mice were euthanized and tumor tissue was fixed in 10% buffered formalin for 48 h or flash frozen on a dry ice ethanol bath and stored at −80°C. For studies involving treatments, mice were randomized into treatment arms when the average tumor size of the entire cohort reached ~150mm3. Lapatinib was purchased from Glaxosmithkline (TM Tykerb), tablets pulverized and administered as a suspension in 0.5% hydroxypropylmethylcellulose and 0.2% Tween 80 by oral gavage in two daily doses for a total of 40 mg/day of lapatinib.

#### Protein purification and crystallography

The human HER3 kinase domain fragment, residues 674-1001 (numbering shown with/without the 19-aa signal sequence) was expressed in SF9 cells using the Bac-to-Bac expression system and purified as previously described (Jura et al., 2009b). The construct contained N-terminal polyhistidine tag, which was cleaved prior to crystallization. The HER3/bosutinib complex was formed by dilution of DMSO-solubilized bosutinib into crystallization buffer (10 mM Tris pH 8.0, 150 mM NaCl, 1 mM DTT, 1 mM TCEP) to a final concentration of 210 uM. HER3 kinase domain was then added to a final concentration of 6 mg/mL (160 uM). The complex was crystallized in the hanging drop format by diluting the above solution with an equal volume of mother liquor containing 100 mM MES pH6.7, 10% PEG 20,000. Crystals were cryoprotected by soaking in a solution containing mother liquor plus 30% glycerol, then flash frozen in liquid nitrogen. Diffraction data was collected on beamline 8.3.1 of the Advanced Light Source at the Lawrence Berkeley National Laboratory. Data processing was completed using mosflm and scala. Two datasets were merged to obtain greater completeness in the high resolution shells. The crystal structure was determined by molecular replacement using a structure of the HER3 kinase domain bound to AMP-PNP (PDB ID 3KEX) after removal of the ligand (Jura et al., 2009b). A positive electron density corresponding to bosutinib was visible in the ATP binding site immediately after molecular replacement. After two rounds of manual model building and automated refinement using COOT and Phenix, respectively, bosutinib was built into the positive density and the structure was refined to completion (Adams et al., 2010; Emsley et al., 2010). Detailed statistics for data collection and refinement can be found in Table S1.

#### MD simulations

Simulation systems were prepared by placing the HER3 KD in either the presence or absence of bound ATP (based on PDB ID: 3KEX (Jura et al., 2009b)) in a cubic simulation box with periodic boundary conditions (~81 Å per side, containing ~55,000 atoms). Waters were represented explicitly, and Na^+^ and Cl^−^ ions were added to neutralize the system and achieve physiological salinity of 150 mM. The systems were parameterized with the Amber99SB*-ILDN (Best and Hummer, 2009; Hornak et al., 2006; Lindorff-Larsen et al., 2010) force field with TIP3P water (Jorgensen et al., 1983), and Amber parameters for ATP were used with torsion coefficients obtained from fitting to quantum calculations. The systems were each equilibrated on GPU Desmond using a mixed NVT/NPT schedule (Bergdorf et al., 2015). MD simulations were performed on the special-purpose machine Anton (Shaw et al., 2014) in the NPT ensemble with T = 310 K and P = 1 bar using a variant (Lippert et al., 2013) of the Nosé-Hoover (Hoover, 1985) and the Martyna-Tobias-Klein algorithm (Martyna et al., 1994). The simulation time step was 2 fs; the r-RESPA integration method was used, with long-range electrostatics evaluated every three time steps. The electrostatic forces were calculated using the *u*-series method (Predescu et al., 2019) with a 1.37-nm cutoff for the electrostatic pairwise summation. A 9-Å cutoff was applied for the van der Waals calculations.

Mutual information between residues making up the AP-2 pocket (transmitter) and the ATP-binding site (receiver) for the WT HER3 kinase domain were calculated using N-body Information Theory (NbIT) (LeVine and Weinstein, 2014). The AP-2 pocket was defined as a selection of the residues 683, 685, 689, 690, 709, 718, 720, 756, and 767, and the ATP-binding as the residues of the P loop (696–704). The NbIT calculation was performed on three representative 10 μs trajectories, and only the Cα and Cβ atoms of each residue were included the calculation.

#### Unbiased MD simulations of HER3 interacting with chemical fragments

These simulations were similar to the previously reported simulations of small-molecule binding of Src kinase (Shan et al., 2011), in that the protein and the small molecules were not subjected to any biasing force. The HER3 simulation system, containing ~55,000 atoms, was prepared with a similar protocol to that described above. 16 fragments containing aromatic rings and ranging between 200 and 300 kDa with logP <3 were randomly selected from the eMolecules database; these fragments were placed at random positions away from the protein. Two 10-μs unbiased simulations were performed independently in the presence of bound ATP and Mg at the orthosteric site.

### QUANTIFICATION AND STATISTICAL ANALYSIS

The quantitative mouse tumor volume data is shown as the mean value with error bars showing the standard error of the mean (SEM). The sample sizes are shown below each graph including the beginning sample size and the surviving sample size over the time course of the experiments. The graphs were plotted using Excel. Statistics for the crystal structure analysis are provided in Table S1.

## Supplementary Material

1

## Figures and Tables

**Table:** KEY RESOURCES TABLE

REAGENT or RESOURCE	SOURCE	IDENTIFIER
Antibodies		
Anti-HER2	SantaCruz Biotechnology	C-18 #284
Anti-HER3	SantaCruz Biotechnology	5A12 #81455
Anti-HER3 biotin	SantaCruz Biotechnology	C-17/custom
Anti-myc	SantaCruz Biotechnology	9E10 #40
Anti-actin	SantaCruz Biotechnology	I-19 #1616
Anti-HA	SantaCruz Biotechnology	Y11 sc-805
Anti-HA	SantaCruz Biotechnology	F-7 sc-7392
Anti-p-HER3 Y1289	Cell Signaling Technology	4791
Anti-p-HER2 Y1248	Cell Signaling Technology	2247
Anti-myc	Cell Signaling Technology	71D0 #2278
Anti-streptavidin	Cell Signaling Technology	3999
Anti-Akt	Cell Signaling Technology	4058
Anti-phospho-Akt	Cell Signaling Technology	9271
Anti-MAPK	Cell Signaling Technology	9102
Anti-phospho-MAPK	Cell Signaling Technology	9101
Anti-Flag	Clontech	635691
Anti-rabbit IgG-HRP	GE Healthcare	NA9340
Anti-mouse IgG-HRP	Cell Signaling Technology	7076
Anti-goat IgG-HRP	SantaCruz Biotechnology	2020
Anti-mouse IgG Alexa 546	Invitrogen	A11630
Chemicals, peptides, and recombinant proteins		
Lapatinib	GlaxoSmithKline	Tykerb
Fetal bovine serum	Gemini Bioproducts	100-106
Penicillin-Streptomycin-Glutamine	ThermoFisher	10378016
Heregulin β1	SigmaAldrich	H0786
Phosphatase inhibitor cocktail	Roche	04906845001
Lipofectamine 2000	ThermoFisher	11668500
JetPrime transfection reagent	Polyplus	101000015
Transdux reagent	SystemsBio	LV850A-1
Optimem media	ThermoFisher	31985070
Protein G Sepharose 4 fast flow	GE Healthcare	17-0618-02
Streptavidin conjugated agarose	SigmaAldrich	85881
Streptavidin HRP	Cell Signaling Technology	3999
ProLong Gold antifade reagent	Life Technologies	P36930
Hoechst 33342	ThermoFisher	62249
VectaShield anti-fade with DAPI	Vector Laboratories	H-1200
SNAP Cell 647-SiR	New England Biolabs	S9102S
Paraformaldehyde	Alfa Aesar	43368
Transdux reagent	SystemsBio	LV850A-1
Puromycin	ThermoFisher	A1113802
Critical commercial assays		
Pierce BCA assay	ThermoFisher	23227
QuikChange II Site-directed mutagenesis kit	Stratagene	200521
Deposited data		
HER3 KD bound to AMP-PNP	Protein Data Bank	PDB: 3KEX
Experimental models: Cell lines		
Hamster: CHO-K1 Chinese hamster ovary cells	ATCC	CCL-61
Human: SkBr3 breast cancer cells	ATCC	HTB-30
Human: HCC1569 breast cancer cells	ATCC	CRL-2330
Experimental models: Organisms/strains		
NSG (NOD-scid IL2Rgamma^null^) mice	Jackson Labs	005557
Recombinant DNA		
pDONR221	ThermoFisher	12536017
pENTR4	ThermoFisher	A10465
pDONR223-HER3	gift from William Hahn & David Root - Addgene	23874
pDEST40-2XFL	ThermoFisher/modified	12274015
pDEST40-2XHA	ThermoFisher/modified	12274015
pDEST40-nSNAP2XHA	ThermoFisher/modified	12274015
pDEST40-cSNAP2XFL	ThermoFisher/modified	12274015
pLEX-IRES-GFP destination	modified from pLEX307, gift of David Root, Addgene	41392
Software and algorithms		
Visual Molecular Dynamics	http://www.ks.uiuc.edu/Research/vmd/	Version 1.9.3
PyMol software	https://pymol.org/2/	Version 2.3
The molecular dynamics (MD) simulations were performed using the Anton 2 supercomputer. The simulation code we used is specialized to Anton 2, but codes for performing MD simulation are widely available.		N/A
Other		
Glass slides	ThermoFisher	12-550-15
Bac to Bac Baculovirus Expression System	ThermoFisher	10359016

## Highlights

- HER3 allosteric function is critical for HER2-amplified cancer growth
- The HER2 kinase receiver interface is a potential target site for therapeutics
- The HER3 ATP binding site occupancy is required for activation function
- The HER3 AP-2 pocket is a targetable site that is in cross-talk with the ATP pocket
