# Supplementary material for: Targetable HER3 functions driving tumorigenic signaling in HER2-amplified cancers
Source: Cell Rep. Author manuscript; Available in PMC 2022 Mar 2. (PMC8889928; doi:10.1016/j.celrep.2021.110291)
Supplement: 1 [file NIHMS1776708-supplement-1.pdf]

Cell Reports, Volume *38*

Supplemental information

Targetable HER3 functions driving tumorigenic signaling in HER2-amplified cancers

Marcia R. Campbell, Ana Ruiz-Saenz, Elliott Peterson, Christopher Agnew, Pelin Ayaz, Sam Garfinkle, Peter Littlefield, Veronica Steri, Julie Oeffinger, Maryjo Sampang, Yibing Shan, David E. Shaw, Natalia Jura, and Mark M. Moasser

A

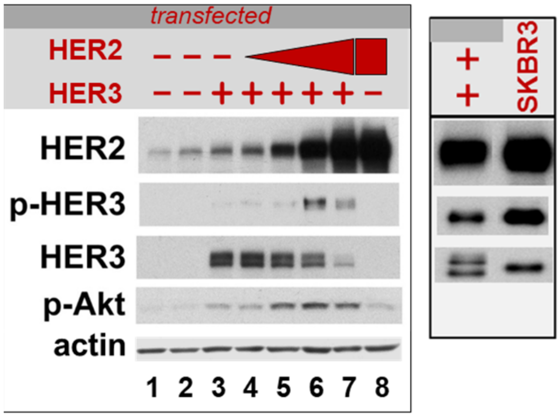

B

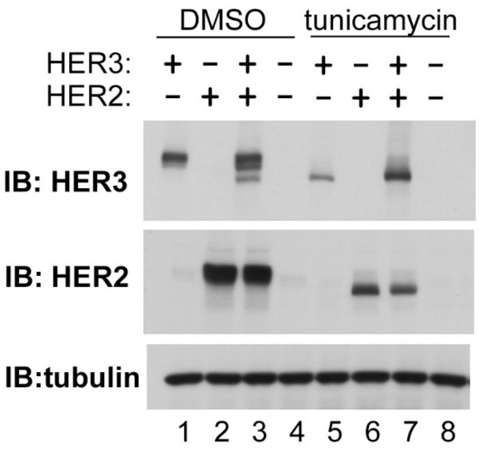

Figure S1. Experimental induction of HER2 overexpression in CHO cells. Related to figures 1,3.
A) CHO cells were transfected with increasing amounts of pDEST40-HER2 and a fixed amount of pDEST40-HER3 and HER3 phosphorylation was assayed by western blotting following 12 hours of serum starvation as shown. The analysis shows constitutive HER3 phosphorylation at high levels of HER2 expression. The vector concentrations and expression levels of lane 6 were selected for the structure-function studies. The blot on the right shows the expression level of these transfected CHO cells side-by-side with lysates from HER2-amplified breast cancer cells.
B) CHO cells were transfected with pDEST40-HER2 and a pDEST40-HER3 at previously established expression levels. Cells were treated with 1ug tunicamycin or control overnight and the cell lysates immunoblotted to observe HER2 or HER3 expression and migration sizes. When high levels of HER2 are co-expressed, HER3 migrates as a doublet in these cells (lane 3). This is due to glycosylation differences that become much more disparate only when HER2 is overexpressed. This is confirmed here by tunicamycin treatment which inhibits glycosylation and the associated double banding of HER3 (lane 7).

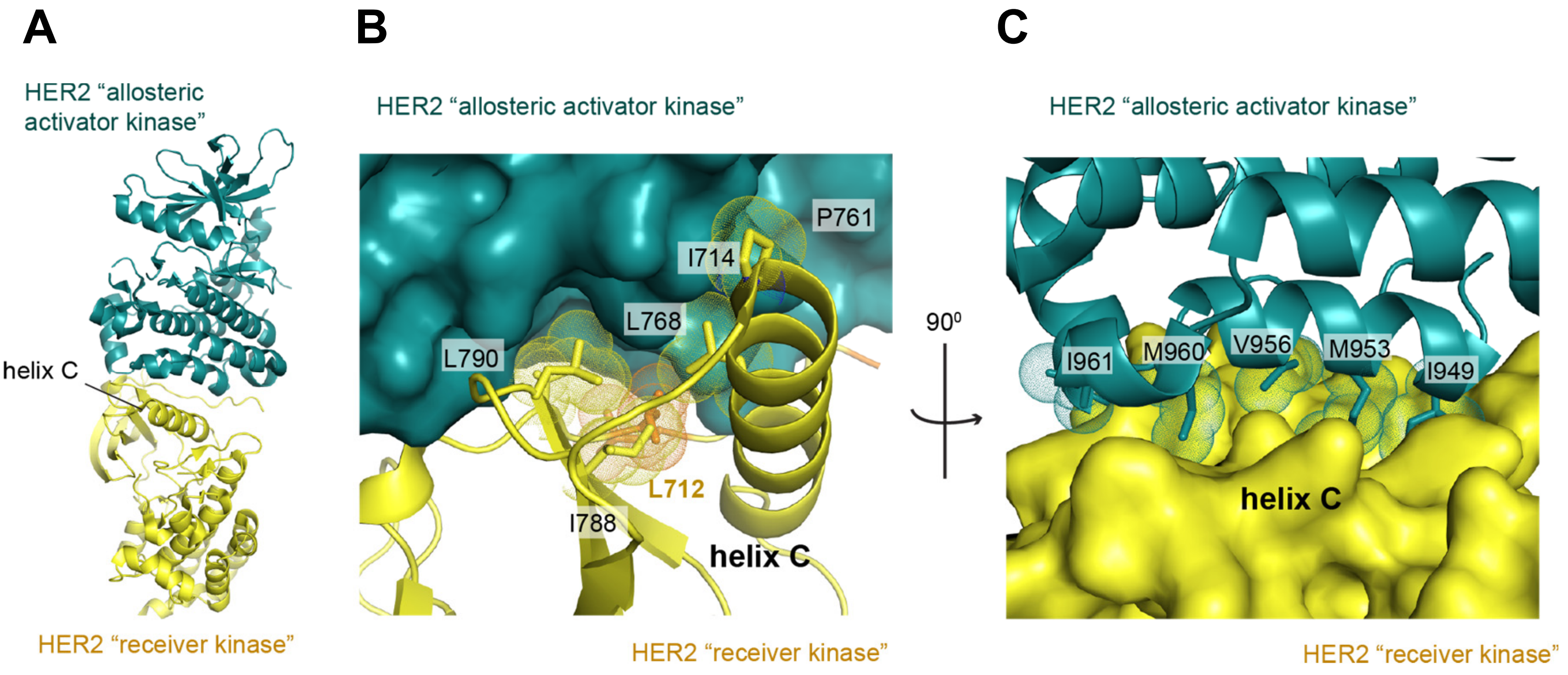

Figure S2. The receiver interface of the HER2 kinase domain. Related to figure 1.
A) The cartoon representation of the HER2 asymmmetric kinase dimer present in the crystal lattice of the HER2 kinase domain structure in complex with the dual HER2/EGFR kinase inhibitor, TAK-285 (PDB: 3PP0). **B).** Close-up view of the receiver interface located within the kinase N-lobe depicts hydrophobic residues that contribute to the asymmetric dimer interface in the receiver. Residues that were mutated in HER2 to their equivalents in the EGFR structure are colored in orange. This was necessary to enable HER2 crystallization. **C).** Close-up view of the activator interface located within the C-lobe depicts hydrophobic residues that contribute to the asymmetric dimer interface in the activator.

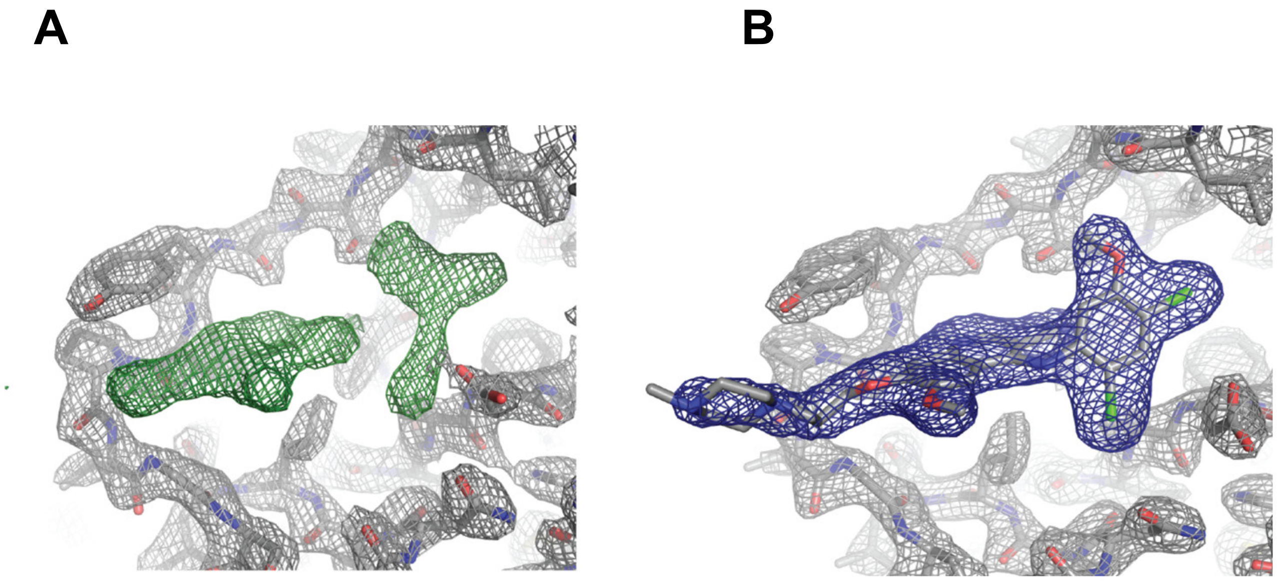

Figure S3. Electron density for bosutinib in the HER3/bosutinib crystal structure. Related to figure 2.
A) Positive electron density (green, Fo-Fc, 2.5σ) observed in the HER3 ATP-binding site after molecular replacement with a ligand-free HER3 kinase domain structure. **B)** Electron density (blue, 2Fo-Fc, 1.5σ) for bosutinib in the refined structure. Density for HER3 residues is shown in grey (2Fo-Fc, 1.5σ).

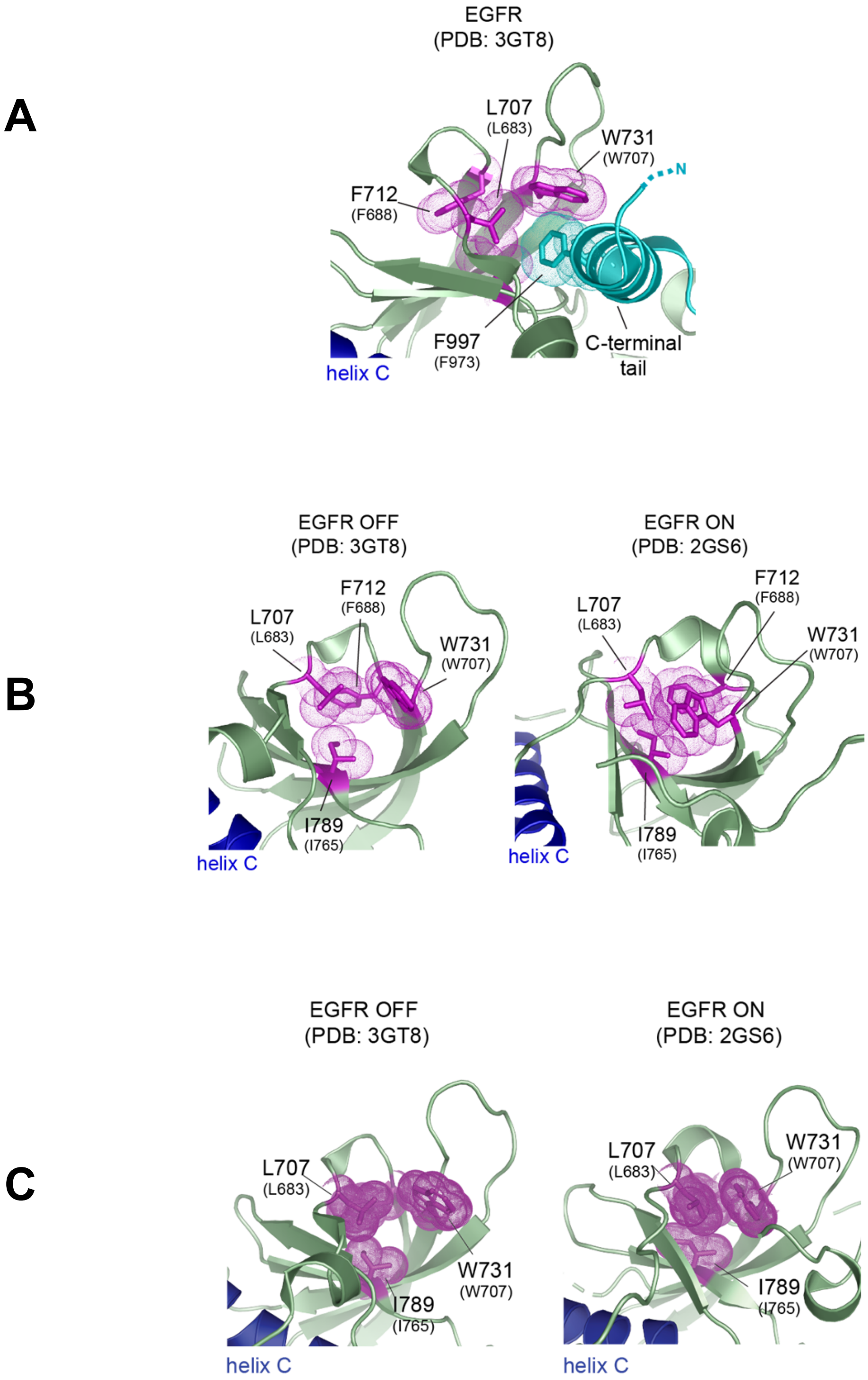

Figure S4. The AP2 pocket on the active and inactive conformations of the EGFR kinase domain. Related to figures 2,3.

A) The AP-2 pocket of inactive EGFR (PDB: 3GT8) is occupied by the AP-2 motif in the C-terminal tail. The side chain of F997 within the C-terminal tail forms an edge-to-face π-stacking interaction with the W731 side chain in the AP-2 pocket. **B-C)** Comparison between the conformations of the AP-2 pocket in the structure of the inactive (PDB: 3GT8) and active (PDB: 2GS6) EGFR kinase illustrates a transition from the open conformation in the inactive kinase that can accommodate ligands, as illustrated in (A) to a closed conformation in the active kinase domain. The comparison is presented using two different alignment modes: using the kinase C-lobes (B) and kinase N-lobes (C). In each case, the AP-2 pocket is in a more closed conformation in the active EGFR kinase.

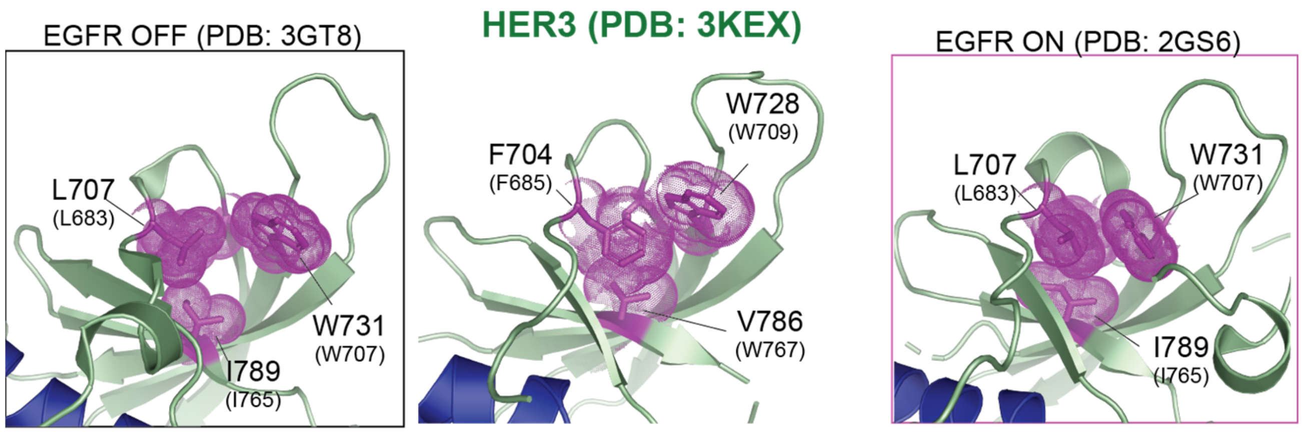

Figure S5. The AP2 pocket in the HER3 and EGFR kinase domains. Related to figures 2,3. Comparison of the AP-2 pocket conformation as seen in the crystal structure of the HER3 pseudokinase domain (PDB: 3KEX, middle panel) with the inactive EGFR kinase domain (PDB: 3GT8, left panel) and the active EGFR kinase domain (PDB: 2GS6, right panel).

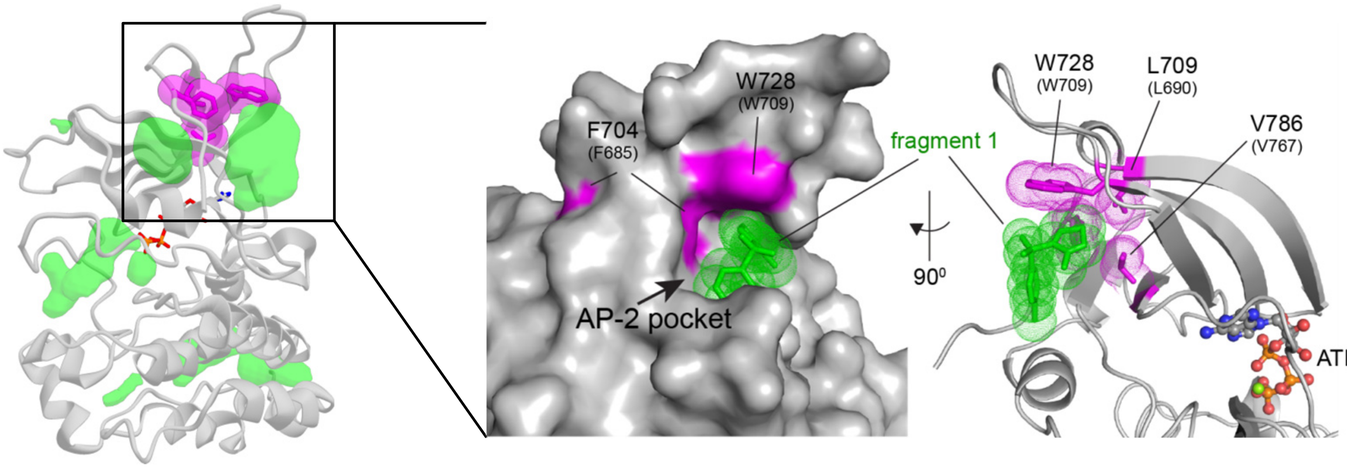

Figure S6. The ability of the HER3 AP2 pocket to engage small molecules. Related to figure 3. (Left) Average occupancy for small molecule fragments (depicted in green) on the HER3 pseudokinase domain identified by unbiased fragment-binding molecular dynamics simulations. The AP-2 pocket is highlighted in purple for reference. (Middle) The zoomed in view of the AP-2 pocket of HER3 in surface representation colored in gray is shown transiently bound with a small molecule fragment in sticks and dot representation colored in green. (Right) A rotation of the same view, with HER3 shown in cartoon representation. Residues that form the potential binding pocket at the AP-2 site and the transiently bound fragment are shown in sticks and dots representation.

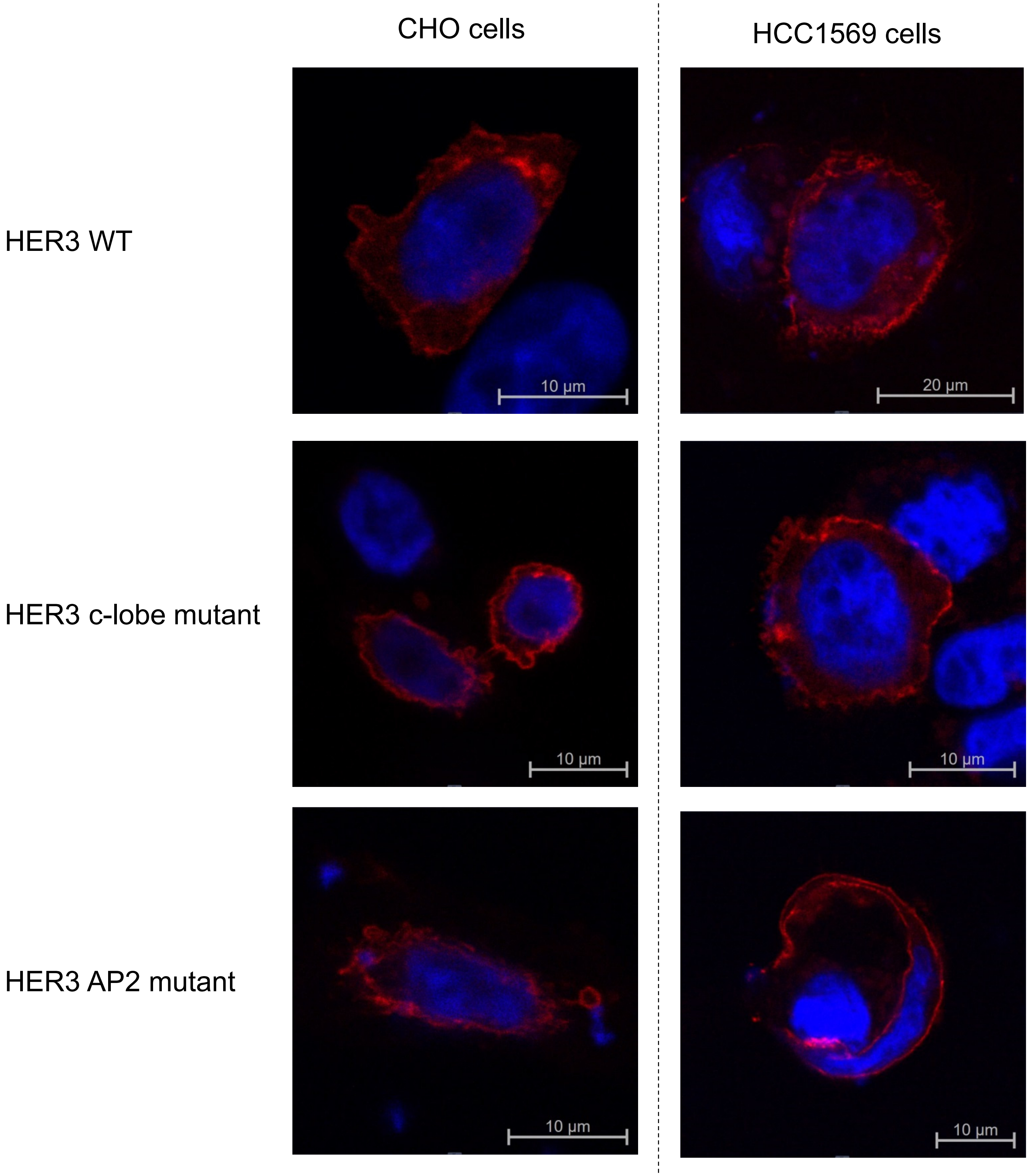

Figure S7. Proper localization of experimental HER3 mutants. Related to figures 1,3,4.
CHO cells (left) or HCC1569 cells (right) were grown on cover slips and transiently transfected with the indicated wildtype or mutant HER3 constructs expressing c-terminal SNAP tags. 24 hours following transfection cells were stained with SNAP-Cell 647-SiR, fixed in paraformaldehyde, and imaged under confocal microscopy. These images were taken from a 1µm thickness z-stack slice across the center of the cell to best demonstrate membrane localization. Cells with more modest expression of the constructs are shown, since massive overexpression creates a non-physiologic localization pattern that is less reliable for demonstrating the functional nature of these mutant and wildtype constructs. Vector transfected controls showed no staining. The HER3 C-lobe mutant is I938R/V945R/M949R. The HER3 AP2 pocket mutant is F704D. Scale bars indicate the size of the field in view.

Table S1. Data collection and refinement statistics. Related to Figure 2.

	**HER3_Bosutinib**
**Wavelength (Å)**	1.1111
**Resolution range**	41.15 - 2.501 (2.591 - 2.501)
**Space group**	P 31 2 1
**Unit cell**	116.385 116.385 58.1941 90 90 120
**Total reflections**	31270 (2524)
**Unique reflections**	15679 (1305)
**Multiplicity**	13.4 (5.6)
**Completeness (%)**	98.10 (83.1)
**Mean I/sigma(I)**	15.5 (2.7)
**Wilson B-factor**	31.95
**R-merge**	0.141 (0.596)
**R-meas**	0.147 (0.654)
**R-pim**	0.039 (0.259)
**CC1/2**	0.996 (0.81)
**CC***	1 (0.962)
**Reflections used in refinement**	15679 (1305)
**Reflections used for R-free**	761 (67)
**R-work**	0.1691 (0.2100)
**R-free**	0.2290 (0.2482)
**CC(work)**	0.960 (0.899)
**CC(free)**	0.937 (0.873)
**Number of non-hydrogen atoms**	2256
**macromolecules**	2130
**ligands**	36

solvent	90
**Protein residues**	277
**RMS(bonds)**	0.007
**RMS(angles)**	0.88
**Ramachandran favored (%)**	97.79
**Ramachandran allowed (%)**	2.21
**Ramachandran outliers (%)**	0.00
**Rotamer outliers (%)**	1.35
**Clashscore**	2.32
**Average B-factor**	32.95
macromolecules	32.78
ligands	34.77
solvent	36.22

Statistics for the highest-resolution shell are shown in parentheses.
